# Health-related quality of life in patients on maintenance hemodialysis: Evidence from southern Iran using EQ-5D-5L and KDQOL-SF

**DOI:** 10.1371/journal.pone.0342155

**Published:** 2026-02-13

**Authors:** Hassan Karami, Neda Mohammadi, Maryam Shirvani Shiri, Najmeh Bordbar, Ali Mouseli, Reihaneh Taheri Kondar, Ali Rezvani, Mobina Vatankhah

**Affiliations:** 1 Social Determinants in Health Promotion Research Center, Hormozgan Health Institute, Hormozgan University of Medical Sciences, Bandar Abbas, Iran; 2 Non-communicable Diseases Research Center, Research Institute for Prevention of Non-Communicable Diseases, Qazvin University of Medical Sciences, Qazvin, Iran; 3 Health Human Resources Research Centre, School of Management and Medical Information Sciences, Shiraz University of Medical Sciences, Shiraz, Iran; 4 Student Research Committee, Faculty of Health, Hormozgan University of Medical Sciences, Bandar Abbas, Iran; 5 Student Research Committee, Faculty of Nursing and Midwifery, Hormozgan University of Medical Sciences, Bandar Abbas, Iran; 6 Student Research Committee, Faculty of Medicine, Hormozgan University of Medical Sciences, Bandar Abbas, Iran; The University of the West Indies, JAMAICA

## Abstract

**Background:**

Hemodialysis (HD) is a common treatment for end-stage renal disease (ESRD) but is often accompanied by markedly reduced health-related quality of life (HRQoL). This study aimed to assess HRQoL and its determinants among HD patients.

**Methods:**

In this cross-sectional study, 203 adult HD patients in Bandar Abbas, Iran, were evaluated. HRQoL was measured via the EQ-5D-5 L index, EQ-VAS, and SF-12 physical (PCS) and mental (MCS) component summary scores. Multivariable linear regression models, identified factors significantly associated with HRQoL.

**Results:**

The mean (SD) EQ-5D-5 L index, EQ-VAS, PCS, and MCS scores were 0.50 (0.47), 64.9 (23.7), 42.3 (8.1), and 42.3 (9.0), respectively. Individuals with higher levels of education demonstrated significantly better EQ-5D-5L index scores (β: 0.24, 95% CI: 0.06 to 0.41 for <6 classes; β: 0.22, 95% CI: 0.03 to 0.40 for 6–12 classes; β: 0.33, 95% CI: 0.13 to 0.52 for >12 years). Being divorced/widowed (β: −20.5, 95% CI: −35.7 to −5.3) or retired/disabled (β: −22.5, 95% CI: −41.6 to −3.3) showed a statistically significant association with reduced EQ-VAS scores. Having supplemental insurance was significantly linked to higher PCS scores (β: 2.4, 95% CI: 0.1 to 4.6), whereas current tobacco use was linked to lower MCS scores (β: −3.7, 95% CI: −7.4 to −0.02). A duration of dialysis ≥5 years was significantly associated with lower EQ-5D-5 L index scores (β: −0.17, 95% CI: −0.31 to −0.04). Comorbidities, age, and sex were not significantly associated with any of the HRQoL measures.

**Conclusion:**

HRQoL among HD patients in southern Iran was markedly reduced and influenced by socioeconomic, lifestyle, and treatment-related factors. Enhancing patient education, expanding insurance coverage, addressing gaps in social support, and incorporating lifestyle interventions—such as smoking cessation—may be associated with modest improvements in HRQoL outcomes in this population.

## 1. Introduction

Chronic kidney disease (CKD) is a progressive condition characterized by a gradual decline in renal function, which may ultimately lead to ESRD, where kidney replacement therapies such as dialysis or transplantation become necessary [1]. CKD can result from various causes, including diabetes, hypertension, and genetic predispositions, and often manifests through symptoms such as fatigue, edema, and fluid imbalance. It frequently involves multiple system complications—most notably cardiovascular disease, bone mineral disorders, and electrolyte imbalances [1]. Owing to its chronic and advancing nature, CKD requires continuous monitoring and comprehensive care to manage symptoms and prevent secondary complications [2].

CKD is recognized as a growing public health challenge worldwide. According to the World Health Organization, approximately 10% of the global population is affected by some degree of CKD, with over two million people currently receiving dialysis [3]. The Global Burden of Disease (GBD) report indicates that in 2019, approximately 8,389,161 individuals in Iran were living with CKD, accounting for nearly 10% of the country’s population in that year, highlighting the significant burden of this disease. [4]. The increasing prevalence of CKD places substantial strain on healthcare systems, particularly due to the long-term demand for complex and costly treatment services [5].

Among kidney replacement modalities, HD remains the most commonly used therapy for ESRD patients worldwide [6]. Typically, HD is performed multiple times per week and involves extracorporeal blood filtration to remove toxins and excess fluids. Despite its widespread availability, HD is associated with considerable physical, psychological, and social burdens. Numerous studies have shown that patients undergoing HD experience high levels of anxiety, depression, and social isolation, all of which can severely diminish quality of life and treatment adherence. In addition, dietary restrictions, limitations in daily functioning, and dependency on healthcare facilities may significantly impact patients’ sense of autonomy and social engagement [1,7].

Although HRQoL has gained increasing attention as a key outcome in chronic disease management, the determinants of HRQoL in patients receiving dialysis remain incompletely understood. Previous research has highlighted the potential influence of variables such as social support, economic status, comorbid conditions, duration of dialysis, and demographic characteristics [8]. A deeper understanding of these factors is essential for guiding patient-centered interventions and improving care outcomes.

Nevertheless, the literature is largely dominated by studies conducted in high-income countries [8–10], and context-specific evidence from low- and middle-income settings such as Iran is lacking. In particular, few studies have employed both generic and disease-specific instruments to assess HRQoL while concurrently examining the combined effects of clinical and individual-level variables. Furthermore, limited data are available from southern Iran, where sociocultural and healthcare delivery contexts may differ significantly from those in other areas. This gap underscores the need for localized evidence to inform evidence-based policy and tailored interventions.

Accordingly, the present study aimed to assess HRQoL among patients undergoing maintenance HD in southern Iran via both a generic (EQ-5D-5 L) instrument and a disease-specific (KDQOL-SF) instrument. The study also examined the associations between HRQoL and patients’ comorbidities. The study also considered demographic and clinical variables to provide a more comprehensive understanding of the factors associated with HRQoL in this population.

## 2. Methods

### 2.1. Study design

This cross-sectional study was conducted between 13 January 2025 and 22 June 2025 in Bandar Abbas County, the capital of Hormozgan Province, southern Iran. According to the 2016 national census, the county’s population was 680,366 [11]. The study followed the STROBE guidelines for reporting cross-sectional research [12].

### 2.2. Setting and eligibility criteria

Patients with end-stage kidney disease (ESKD) undergoing maintenance HD for more than three months were recruited from the dialysis units of Shahid Mohammadi, Khaleej Fars, and the Children’s Hospital in Bandar Abbas. The inclusion criteria were age over 18 years and willingness to participate. The exclusion criteria included age under 18 years, refusal to participate, acute kidney injury, severe cognitive impairment, advanced comorbidities (e.g., class IV heart failure, metastatic cancer, decompensated cirrhosis), or a history of untreated major psychiatric disorders. The average dialysis frequency in the dialysis centers is three sessions per week. The dialyzer filters used are PS130 and PS160, manufactured by Meditechsys (Iran), made of polysulfone, and sterilized with ethylene oxide.

### 2.3. Study outcomes, predictors, and confounders

#### 2.3.1. Outcomes.

The primary outcomes were HRQoL, which was assessed via the EQ-5D-5 L index and the EQ-VAS, PCS and MCS scores. Higher scores reflect better health status.

#### 2.3.2. Predictors and potential confounders.

All socio-demographic, clinical, and lifestyle variables were treated as independent predictors of HRQoL. No variable was classified as a confounder, as the study aimed to assess associations rather than infer causality. Laboratory parameters (e.g., urea, creatinine, and electrolytes) were excluded from the final models because of nonsignificant associations in the preliminary analyses.

### 2.4. Data sources and measurement

#### 2.4.1. Data sources and collection procedures.

Data were obtained from patients’ medical records and through standardized questionnaires administered during dialysis visits. Clinical information covering the preceding three months was extracted from medical files. Access to patient records and interviews was conducted between January 13, 2025, and June 22, 2025. Following coordination with the dialysis centers and explanation of the study objectives to both the centers and the patients, informed consent was obtained from the randomly selected participants. Socio-demographic data and HRQoL information were collected through face-to-face interviews carried out by trained student interviewers, including final-year medical students and master’s-level nursing students. Interviews were conducted from Sunday to Thursday each week, prior to the start of the dialysis session, within the first 45 minutes of treatment. All interviews, regardless of participants’ literacy level, were conducted by trained interviewers and recorded by the interviewers, following standardized procedures, with questions presented orally in an understandable manner and participants’ privacy fully respected. This approach was particularly important given the older age of most participants, who might have faced difficulties with reading, writing, or maintaining concentration for extended periods. The data used in this study are part of a larger research project on HRQoL in patients undergoing maintenance HD. Separate analyses addressing other research questions may be reported in future publications.

#### 2.4.2. Measurement instruments.

**2.4.2.1. Socio-demographic and clinical history:** Socio-demographic data included age, sex, marital status, educational level, place of residence, employment status, and health insurance coverage. The clinical variables included the duration of dialysis, presence of comorbidities, hospitalization within the past year, tobacco use, primary cause of renal failure, and history of kidney transplantation. Data on blood pressure and biochemical markers—including serum creatinine, urea, haemoglobin, haematocrit, potassium, calcium, phosphate, and dialysis adequacy (Kt/V)—were also collected.

**2.4.2.2. Persian version of the euroqol 5-dimensions 5-levels questionnaire (EQ-5D-5 L)** **:** The validated Persian version of the EQ-5D-5 L [13] was used to assess HRQoL across five dimensions: mobility (MO), self-care (SC), usual activities (UA), pain/discomfort (P/D), and anxiety/depression (A/D). Each domain is rated on a five-level scale, from 1 (no problems) to 5 (extreme problems). In MO, SC, and UA, a score of 5 denotes incapacity, whereas in A/D and P/D, it reflects an extreme feeling of distress [14]. Health utility scores were calculated via the Iranian EQ-5D value set developed by Afshari et al. through composite time trade-off (TTO) and discrete choice experiment (DCE) methods [14]. The resulting index ranges from −1.19 (states worse than death) to 1 (perfect health), on the basis of societal preferences [14]. Changes between 0.034 and 0.134 points were considered the minimal clinically important difference (MCID) [15].The EQ-VAS, a visual analogue scale ranging from 0 (worst imaginable health) to 100 (best imaginable health), was also administered as a self-rated measure of perceived health [14].

**2.4.2.3. Persian version of the kidney disease quality of life instrument (KDQOL-SF™ v1.3):** The KDQOL-SF is a 79-item instrument designed to assess HRQoL in patients with kidney disease. It comprises two main components: disease-specific subscales (e.g., symptoms/problems, disease impact, burden, work status, cognitive function, social interactions, sexual function, sleep, social support, staff encouragement, patient satisfaction, and overall health rating) and general health subscales derived from the SF-12 (e.g., physical functioning, pain, general health, vitality, emotional and social functioning, and mental health) [16]. In addition, two composite scores are calculated: the PCS, reflecting physical health status, and the MCS, reflecting emotional well-being [16]. Each domain is scored on a 0–100 scale, with higher scores indicating better HRQoL. Scores are computed on the basis of standardized scoring procedures provided by the KDQOL working group (KDQOL v1.3 template) [16]. The Persian version of the KDQOL-SF, validated by Yekaninejad et al., has demonstrated strong psychometric properties, with Cronbach’s alpha coefficients ranging from 0.73 to 0.93, confirming construct validity [17].

### 2.5. Ethical considerations

Ethical approval was obtained on 10 November 2024 from the Ethics Committee of Hormozgan University of Medical Sciences (IR.HUMS.REC.1403.253). All participants were informed about the objectives and procedures of the study prior to participation. Written informed consent was obtained from all participants before data collection. Participation was entirely voluntary, and respondents were assured of the confidentiality and anonymity of their information. The study did not include individuals under 18 years of age; therefore, no parental or guardian consent was required. The ethics committee did not waive the requirement for consent.

### 2.6. Bias considerations

To minimize bias and enhance validity, a simple random sampling method was used to obtain a representative sample and reduce selection bias. Standardized, validated instruments were employed uniformly to limit information bias. To control for response bias, all the interviews were conducted by three trained interviewers with final-year medical students and master’s-level nursing students. The data collection process was closely supervised to ensure consistency and adherence to the protocol.

### 2.7. Sample size determination and sampling

#### 2.7.1. Descriptive objectives.

The sample size was estimated to determine the mean HRQoL scores (KDQOL-SF, EQ-5D-5 L, and EQ-VAS) with acceptable precision, using data from prior studies as references [18–20]. The arithmetic means of the reported means and standard deviations were calculated. A 10% margin of error relative to the mean was assumed. Calculations were performed via WinPepi (v3.18), which is based on a 95% confidence level, 80% power, and an anticipated 10% dropout rate.

#### 2.7.2. Analytical objectives.

Effect sizes for subgroup analyses based on chronic comorbid conditions were derived from stratified data reported in earlier studies [18,21,22]. Independent t tests and ANOVA were used to compare subgroups on the basis of the data distribution. Sample size estimation for these comparisons was carried out in Stata (v14) via the *sampsi* command, with the largest required sample size determined to be 221 participants.

#### 2.7.3. Sampling procedure.

The sampling procedure involved creating a complete patient list (437 patients) from dialysis units in an Excel file. The participants were then selected through simple random sampling via a random number generator to ensure unbiased recruitment [23].

### 2.8. Handling of quantitative variables

The duration of dialysis was divided into two groups, < 5 years and ≥5 years, to differentiate the short-term and long-term effects on HRQoL. Additionally, to examine differences in HRQoL among patients, age was categorized into three groups: young (<40 years), middle-aged (40–60 years), and elderly (>60 years). These categorizations were selected to create a clear distinction between groups and are based on previous studies [24–27]. Educational level was classified as illiterate, less than 6 classes, 6–12 classes, or more than 12 years. Comorbidities were categorized as 0, 1, or ≥2 conditions. Blood pressure was classified as normal (≤120/80 mmHg), seminormal (121–139/81–89 mmHg), or high (≥140/90 mmHg).

### 2.9. Statistical analysis

Continuous variables are reported as the means ± standard deviations (SDs) if normally distributed and as medians with interquartile ranges (IQRs) otherwise. Categorical variables were summarized using frequencies and percentages. Group comparisons were conducted via independent t tests and one-way ANOVA for normally distributed variables and Mann‒Whitney U or Kruskal‒Wallis tests for non-normally distributed data. Chi-square tests were used to compare EQ-5D dimension responses, dichotomized as “no problem” vs. “any problem”.

Univariate linear regressions were first performed to examine the associations between each independent variable and HRQoL outcomes (EQ-5D index, EQ-VAS, PCS, and MCS). All variables were then included in the initial multivariable models, regardless of univariate significance. The final models were selected using backward elimination based on the Akaike Information Criterion (AIC). This procedure began with full models containing all pre-specified variables and iteratively removed the least contributory variable at each step, continuing only if removal resulted in a lower AIC. The process terminated when no further variable removal could reduce the AIC. To assess the robustness of our model selection, we performed a sensitivity analysis using the Bayesian Information Criterion (BIC), which applies a stronger penalty for model complexity. The consistency of key predictors across both selection approaches supports the stability of our findings. This strategy allowed for the identification of the most relevant predictors while adjusting for all measured covariates. Approximately 3% of the participants had substantial missing data and were excluded from the analyses. Statistical significance was defined as p < 0.05. All analyses were performed via R software (version 4.5.1).

## 3. Results

### 3.1. Recruitment

**Fig 1:**
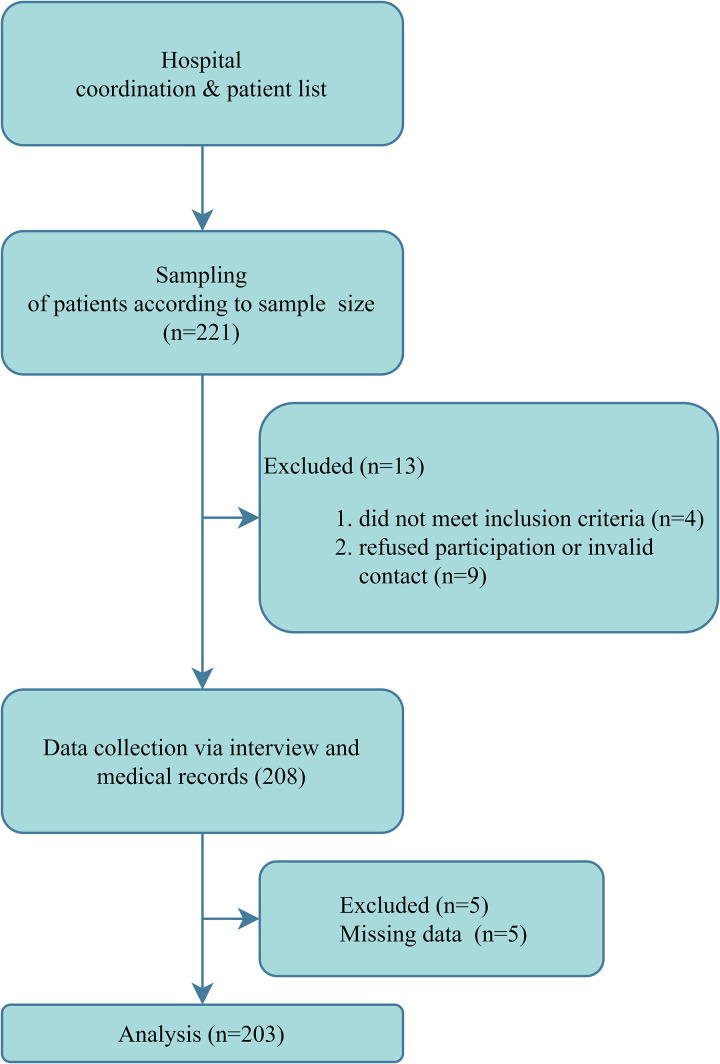
Study flow diagram.

Of 221 eligible patients, 13 were excluded because of ineligibility or refusal, and 5 were excluded because of incomplete data. A total of 203 participants were included in the final analysis (Fig 1).

### 3.2. Patient characteristics

The mean age of the participants was 55.26 years (SD = 15.76), with 41% aged >60 years. Males accounted for 53% of the participants. Most were married (82%), urban residents (71%), and had ≤ 12 years of education (91%). Comorbidities ≥2 were reported in 51% of the patients, and 48% had supplemental insurance. Missing data were minimal (<3%), primarily related to HRQoL and laboratory variables (Table 1).

**Table 1:** Sociodemographic, lifestyle, clinical and biochemical characteristics of the study population (n = 203).

Variables	n (%)/M ± SD	Variables	n (%)/M ± SD
Age	55.26 ± 15.76	Comorbidity	
< 40	38 (18.72)	0	28 (13.79)
40-60	81 (39.9)	1	71 (34.98)
> 60	84 (41.38)	>=2	104 (51.23)
Gender		Main cause of CKD	
Male	107 (52.71)	Not knowing/Unknown	44 (21.67)
Female	96 (47.29)	Diabetes	60 (29.56)
Marital Status		Hypertension	70 (34.48)
Single	18 (8.87)	Others	29 (14.29)
Married	167 (82.27)	Kidney transplant	
Divorced/widowed	18 (8.87)	No	192 (94.58)
Education		Yes	11 (5.42)
Illustrate	55 (27.09)	Past-year hospitalization	
< 6 classes	55 (27.09)	No	55 (27.09)
6–12 classes	75 (36.95)	Yes	148 (72.91)
> 12 classes	18 (8.87)	Dialysis duration (years)	
Occupation		< 5	152 (74.88)
Housekeeper	86 (42.36)	≥ 5	51 (25.12)
Employed	45 (22.17)	Blood pressure	
Disabled/Retried	56 (27.59)	Normal	85 (41.87)
Unemployment	16 (7.88)	Semi-Normal	35 (17.24)
Residence		High	83 (40.89)
Rural	59 (29.06)	Average monthly Kt/V	1.32 ± 0.34
City	144 (70.94)	Serum urea (mmol/L)	67.93 ± 59.89
Sup insurance		Serum creatinine (µmol/L)	67.93 ± 59.89
No	105 (51.72)	Hemoglobin (g/dL)	9.96 ± 2.86
Yes	98 (48.28)	Hematocrit (%)	31.28 ± 6.72
Tobacco use		Potassium (mmol/L)	5.04 ± 1.00
No	124 (61.08)	Serum calcium (mmol/L)	8.61 ± 1.03
Former user	53 (26.11)	Phosphate (mmol/L)	5.27 ± 1.45
Yes	26 (12.81)		

M: mean; SD: Standard Deviation; Sup Insurance: Supplemental Insurance; CKD: ChronicKidney Disease; Blood pressure: Normal (≤120/80 mmHg), Semi-normal (121–139/81–89 mmHg), High (≥140/90 mmHg).

### 3.3. Health-related quality of life scores

#### 3.3.1. EQ-5D-5 L Index.

The mean EQ-5D-5 L index score was 0.50. Scores were statistically and clinically significantly higher among participants aged <40 years (0.67), those employed (0.67), and those without recent hospitalization (0.62). statistically and clinically significantly lower scores were observed among divorced or widowed individuals (−0.05), illiterate individuals (0.21), and those with ≥2 comorbidities (0.37) (Table 2).

**Table 2:** Statistical comparison of mean HRQoL Scores (EQ-5D-5L, EQ-VAS, PCS, MCS) according to sociodemographic and clinical characteristics of study population (n = 203).

Variables	EQ-5D-5L Index	EQ-VAS	PCS	MCS
	Mean ±SD	Mean ±SD	Mean ± SD	Mean ±SD
Total	0.50 ± 0.47	64.94 ± 23.72	42.27 ± 8.05	42.27 ± 8.98
Age				
< 40	0.67^*^ **± 0.43**	65.97 ± 22.00	**43.87 ± 6.81**	39.61 ± 9.83
40-60	0.65 ± 0.33	**70.44 ± 21.44**	44.29 ± 6.99	43.47 ± 8.65
> 60	0.29 ± 0.51	59.17 ± 25.43	39.61 ± 8.82	42.32 ± 8.74
Gender				
Male	0.54 ± 0.47	65.46 ± 24.20	43.09 ± 7.71	42.62 ± 9.24
Female	0.46 ± 0.46	64.36 ± 23.28	41.36 ± 8.37	41.89 ± 8.71
Marital Status				
Single	0.53 ± 0.57	69.31 ± 24.08	43.84 ± 6.78	42.65 ± 11.37
Married	0.56 ± 0.41	66.37 ± 23.13	42.81 ± 7.76	42.60 ± 9.00
Divorced/widowed	−0.05^*^ **± 0.47**	**47.29 ± 22.56**	**35.72 ± 9.28**	38.85 ± 5.01
Education				
Illustrate	0.21^*^ **± 0.54**	**56.76 ± 26.70**	**38.34 ± 9.44**	42.76 ± 8.26
< 6 classes	0.54 ± 0.46	65.51 ± 23.89	43.08 ± 7.73	42.24 ± 9.85
6–12 classes	0.64 ± 0.33	69.55 ± 18.71	44.79 ± 6.25	41.43 ± 9.14
> 12 classes	0.75 ± 0.26	68.98 ± 27.47	41.29 ± 6.82	44.39 ± 7.79
Occupation				
Housekeeper	0.49 ± 0.43	67.93 ± 20.59	41.80 ± 8.35	42.69 ± 8.89
Employed	0.67^*^ **± 0.38**	66.11 ± 26.08	44.44 ± 6.90	40.45 ± 9.75
Disabled/Retried	0.35 ± 0.56	58.18 ± 26.47	41.28 ± 9.06	43.34 ± 9.00
Unemployment	0.63 ± 0.34	69.21 ± 19.11	42.18 ± 4.33	41.41 ± 6.77
Residence				
Rural	0.50 ± 0.47	63.72 ± 24.56	41.49 ± 7.68	41.36 ± 9.83
City	0.51 ± 0.47	65.44 ± 23.43	42.59 ± 8.21	42.64 ± 8.61
Sup insurance				
No	0.49 ± 0.49	63.81 ± 23.10	41.38 ± 8.34	40.90 ± 9.53
Yes	0.52 ± 0.44	66.15 ± 24.43	43.23 ± 7.66	**43.75 ± 8.14**
Tobacco use				
No	0.54 ± 0.44	64.78 ± 23.83	42.71 ± 7.80	43.42 ± 8.91
Former user	0.42 ± 0.50	64.88 ± 25.88	41.35 ± 8.84	41.36 ± 8.38
Yes	0.53 ± 0.50	65.83 ± 18.93	42.06 ± 7.69	**38.67 ± 9.67**
Comorbidity				
0	0.68 ± 0.34	73.66 ± 18.53	46.07 ± 7.02	39.37 ± 9.19
1	0.64 ± 0.35	69.46 ± 19.57	43.43 ± 7.25	42.52 ± 8.77
>=2	0.37^*^ **± 0.52**	**59.51 ± 26.24**	**40.46 ± 8.39**	42.88 ± 9.00
Main cause of CKD				
Not knowing/Unknown	0.57 ± 0.44	62.19 ± 24.93	41.59 ± 8.88	40.18 ± 8.72
Diabetes	0.49 ± 0.47	64.04 ± 26.00	41.35 ± 7.59	41.01 ± 9.00
Hypertension	0.49 ± 0.45	65.39 ± 21.86	42.60 ± 7.25	44.36 ± 9.13
Others	0.47 ± 0.54	69.89 ± 21.49	44.43 ± 9.39	43.02 ± 8.20
Kidney transplant				
No	0.50 ± 0.47	65.13 ± 23.87	42.21 ± 8.09	42.18 ± 9.02
Yes	0.56 ± 0.49	61.56 ± 21.55	43.42 ± 7.58	43.95 ± 8.48
Past-year hospitalization				
No	0.62^*^ **± 0.39**	**70.20 ± 21.29**	43.67 ± 7.48	41.93 ± 10.11
Yes	0.46 ± 0.49	62.99 ± 24.34	41.75 ± 8.22	42.40 ± 8.56
Dialysis duration (years)				
< 5	0.56^*^ **± 0.42**	65.66 ± 22.30	41.88 ± 7.70	42.54 ± 8.49
≥ 5	0.35 ± 0.55	62.80 ± 27.65	43.42 ± 9.01	41.47 ± 10.36
Blood pressure				
Normal	0.57 ± 0.44	69.40 ± 22.18	43.43 ± 7.18	42.25 ± 9.25
Semi-Normal	0.45 ± 0.45	65.92 ± 25.31	42.32 ± 7.38	43.33 ± 8.94
High	0.46 ± 0.49	**59.96 ± 23.88**	41.06 ± 9.03	41.85 ± 8.78

EQ-5D Index: EQ-5D-5L Utility Index Score; EQ-VAS: EuroQol Visual Analogue Scale; PCS: SF-12 Physical Component Summary Score; MCS: SF-12 Mental Component Summary Score; Sup insurance: Supplemental Insurance; CKD: Chronic Kidney Disease; Blood pressure: Normal (≤120/80 mmHg), Semi-normal (121–139/81–89 mmHg), High (≥140/90 mmHg). Statistical tests used: independent samples t-test; one-way ANOVA; and/or Mann –Whitney U test where appropriate. **Bold values** p < 0.05; ^*^ Clinically meaningful.

#### 3.3.2. EQ-VAS score.

The mean EQ-VAS score was 64.9. Scores were significantly higher among individuals aged 40–60 years (70.4) and those without recent hospitalization (70.2). Significantly lower scores were observed among divorced or widowed individuals (47.3), illiterate individuals (56.8), and those with ≥2 comorbidities (59.5) (Table 2).

#### 3.3.3. PCS and MCS scores.

The mean PCS and MCS scores were both 42.3. The PCS score was significantly greater among participants aged <40 years (43.9) and significantly lower among divorced or widowed individuals (35.7), illiterate individuals (38.3), and those with ≥2 comorbidities (40.5). MCS was significantly higher among those with supplemental insurance (43.8) and significantly lower among current tobacco users (38.7) (Table 2).

Among the KDQoL-SF subscales, the lowest mean scores were observed for work status (30.8) and burden of kidney disease (39.7). The highest scores were reported for dialysis staff encouragement (82.6) and social support (79.72) (S1Table). The most frequently reported EQ-5D-5 L problems across the sample were in the domains of P/D (61.6%), anxiety/depression (54.7%), and mobility (54.7%) (S2 Table).

### 3.4. Predictors of health-related quality of life

According to the initial regression model, individuals with fewer than six years of education exhibited a statistically significant increase of 0.22 in EQ-5D index scores (95% CI: 0.01 to0.43). Dialysis duration was significantly associated with a decrease of 0.17 in EQ-5D index scores (95% CI: −0.34 to −0.01). In addition, being disabled or retired was associated with a statistically significant reduction of 26.14 points in EQ-VAS scores (95% CI: −50.11 to −2.17). Unemployment was significantly associated with a 4.72point decrease in Physical Component Summary (PCS) scores (95% CI: −8.65 to −0.79), whereas having supplemental insurance was associated with a statistically significant increase of 2.62 points in PCS scores (95% CI: 0.34 to4.89) (S3 Table). All reported associations were statistically significant.

To improve model parsimony, a final model was selected using the Akaike information criterion (AIC) (Table 3). In this model, all education levels remained positively associated with the EQ-5D scores. EQ-VAS scores were significantly negatively associated with divorced (β = −20.5, 95% CI = [−35.7 to −5.3]) and retired or disabled status (β = −22.5, 95% CI = [−41.6 to −3.3]). The PCS was significantly positively associated with having supplemental insurance (β = 2.4, 95% CI = [0.11 to 4.6]), whereas the MCS was significantly negatively associated with current tobacco use (β = −3.7, 95% CI = [−7.4 to −0.02]).

**Table 3:** Final multivariate regression model selected by akaike information criterion (AIC) (n = 203).

Variables	EQ-5D-Index β (95% CI)	EQ-VAS β (95% CI)	PCS β (95% CI)	MCS β (95% CI)
Age	−0.01 (−0.01to 0)		−0.06 (−0.17to 0.06)	0.08 (−0.02 to 0.18)
Gender (Male)	**Reference**			
Female		−16.53 (−35.03 to 1.96)		
Marital Status (Single)	**Reference**			
Married	0.13 (−0.19 to0.45)	−2.33 (−14.24 to 9.59)	0.65 (−3.81 to5.11)	−0.58 (−7.85 to 6.69)
Divorced/widowed	−0.33 (−0.74 to 0.07)	**−20.52 (−35.71 to−5.33)**	−4.23 (−10.49 to 2.02)	−5.45 (−14 to 3.1)
Occupation (housekeeper)	**Reference**			
Employed		−17.64 (−37.22 to 1.94)		
Disabled/Retried		**−22.45 (−41.58 to −3.32)**		
Unemployment		−18.41 (−37.44 to 0.62)		
Education (Illustrate)	**Reference**			
< 6 class	**0.24 (0.06 to0.41)**		3.55 (−0.28 to 7.37)	
6–12 class	**0.22 (0.03 to 0.40)**		3.79 (−0.21 to 7.78)	
> 12 class	**0.33 (0.13 to 0.52)**		0.32 (−4.65 to 5.29)	
Sup insurance (No)	**Reference**			
Yes			**2.37 (0.11 to 4.63)**	2.17 (−0.35 to 4.69)
Tobacco use (No)	**Reference**			
Former user				−1.87 (−4.7 to 0.96)
Yes				**−3.72 (−7.41 to −0.02)**
Comorbidity (0)	**Reference**			
Comorbidity (1)	0.06 (−0.1 to 0.22)	−3.33 (−12.66 to 5.99)	−1.13 (−4.85 to 2.59)	
Comorbidity (>=2	−0.07 (−0.25 to 0.11)	−9.77 (−19.65 to 0.11)	−3.35 (−7.22 to 0.52)	
Dialysis Duration	**−0.17 (−0.31 to −0.04)**			

EQ-5D Index: EQ-5D-5L Utility Index Score; EQ-VAS: EuroQol Visual Analogue Scale; PCS: SF-12 Physical Component Summary Score; MCS: SF-12 Mental Component Summary Score; Sup insurance: Supplemental Insurance; CI: Confidence interval; **Bold values** p < 0.05.

## 4. Discussion

This study demonstrated that HRQoL was low among patients undergoing HD, with a mean EQ-5D-5 L index of 0.50, an EQ-VAS score of 64.9, and both physical and mental component scores (PCS and MCS) averaging 42.3. In the final multivariable regression model optimized using the AIC, higher educational attainment was significantly associated with better EQ-5D index scores, whereas a dialysis duration of five years or more was significantly associated with lower scores. In addition, being divorced or widowed and being retired or disabled were significantly associated with lower EQ-VAS scores. Having supplemental insurance was significantly associated with higher PCS scores, whereas current tobacco use was significantly associated with lower MCS scores.

### 4.1. Comparisons with previous studies and interpretation

The mean HRQoL in the present study was low among HD patients (EQ-5D-5L index: 0.50; EQ-VAS: 64.9; PCS/MCS: 42.3). Comparable findings were observed across the MENA region, where HRQoL ranged from moderate to poor, including Palestine (0.37; 59.4) [21], Jordan (0.44; 62.1) [28], Saudi Arabia (36.5 VAS) [29], and Turkey (66.7 VAS) [30]. In Asia, South Korea reported relatively higher PCS and MCS scores (61.9; 55.5) [31], while Japan showed pronounced physical impairment (37.1) with similar mental levels (49.6) [32]. Africa and the United States demonstrated substantial variability (South Africa: PCS 65.4, MCS 74.6; United States: PCS 48.8, MCS 51.7) [33,34], whereas Latin American countries reported notably higher HRQoL (Colombia EQ-5D-5L: 0.9; VAS 69.6; Vietnam 0.766) [35,36]. These cross-country differences likely reflect heterogeneity in healthcare systems, the extent of financial coverage for HD, and socio-cultural contexts. Available evidence suggests that stronger universal health and social support is associated with a lower economic and psychological burden of disease and higher HRQoL [37]; therefore, cross-country comparisons should be interpreted within the context of health policies and socio-economic conditions.Furthermore, Mean EQ-5D index scores were higher among younger patients (<40 years), those who were employed, patients without hospitalization in the past year, and those with a dialysis duration of less than five years. In contrast, patients with two or more comorbidities, illiterate individuals, and those who were divorced or widowed had lower scores. These differences were both statistically and clinically meaningful, exceeding the MCID for the EQ-5D index. These findings indicate that HRQoL results from the interaction between clinical factors and social determinants of health and emphasize the need for interventions that go beyond purely clinical care.

The final multivariate regression model showed that compared to illiterate individuals, those with <6, 6–12, and >12 years of education had 0.24, 0.22, and 0.33 higher EQ-5D index scores, respectively, which were statistically significant and clinically meaningful. Similarly, in China, patients with higher education levels reported significantly better HRQoL, a difference attributed to greater disease awareness and broader social support [20]. In Palestine, university education was significantly associated with higher EQ-5D and EQ-VAS scores, with health literacy and active participation in disease management identified as key contributing factors [21]. In the Netherlands, higher education was significantly linked to better HRQoL, which has been explained by more favourable socioeconomic conditions, healthier lifestyles, and greater access to healthcare services [38]. In Iran, a significant association between education level and the mental health status of dialysis patients has also been reported, an association that may partly reflect differences in coping abilities among individuals with higher levels of education. The observed differences in the association between education and HRQoL among HD patients across countries likely reflect heterogeneity in healthcare systems, health literacy levels, cultural norms, and socio-economic conditions [39].

Each additional year of dialysis duration was associated with a 0.17 decrease in EQ-5D-Index, which was statistically significant and clinically meaningful. A similar association was reported in Japan, although the effect size was considered mild, and the reduction was attributed to psychological adaptation, life satisfaction, and the structural support of the healthcare system [10]. In Pakistan, extended time on dialysis was linked to a significant and substantial decline in physical, mental, social, and environmental functioning, which was explained by chronic stress due to repetitive treatments, functional limitations, and impaired social interactions [40]. In China, more years of dialysis were significantly associated with lower KDQOL-36 scores [41]. In contrast, a study from Poland reported no significant association between dialysis duration and patients’ HRQoL [42]. Similarly, an Iranian study reported no statistically significant difference in HRQoL scores between patients who had received dialysis for less than or more than 36 months [43]. The observed differences in the association between dialysis duration and HRQoL are likely due to heterogeneity in healthcare systems, access to support, psychological adaptation, and management of patient complications, and may also be influenced by patients’ mean age. The results show that divorced or widowed individuals scored 20.52 units lower on the EQ-VAS than single individuals, with statistical significance. In China, a significant relationship was also observed between marital status and HRQoL [44]. In Iran, married individuals reported significantly higher scores than single individuals did, a difference attributed to greater social support and higher psychological resilience among those who are married [18]. In contrast, no significant associations between marital status and HRQoL were reported in studies from Palestine and Pakistan [21,45]. These findings suggest that the effect of marital status on HRQoL may be partially influenced by social support, psychological resilience, and cultural context, which may explain the observed cross-country differences [46]. Disabled or retired individuals scored 22.45 units lower on the EQ-VAS than housekeepers, with statistical significance. In Kazakhstan, employed patients appeared to have higher HRQoL in both physical and mental health domains, which may be related to better physical functioning and a more positive mood [47]. Similarly, a study in India showed that employed individuals had higher scores across physical, psychological, and environmental domains—differences that were attributed to financial independence, greater mobility, and fewer limitations in daily activities [48]. In Northern Cyprus, employment was identified as a significant and independent predictor of QoL, with unemployed patients reporting lower scores across most KDQOL-36 dimensions; this disparity was linked to financial dependency, social isolation, and negative psychological consequences among unemployed individuals [49]. In contrast, a study from Bahrain reported no significant association between employment status and QoL among HD patients [50]. This difference between countries may be due to variations in insurance coverage, socio-economic support, cultural norms, rehabilitation programs, and access to supportive services; in Bahrain, comprehensive universal insurance may mitigate financial dependency, social isolation, and the psychological consequences of unemployment, whereas in other countries, employment may enhance quality of life to some extent by providing financial independence, physical mobility, social interaction, and a sense of purpose.

Individuals with supplementary insurance had a 2.37 higher PCS than those without, with statistical significance. Similarly, a study conducted in Nepal demonstrated that patients with health insurance reported higher scores in both physical and mental components of quality of life (PCS and MCS), a difference attributed to reduced treatment-related expenses and greater independence in accessing healthcare services [51]. Findings from a study in China corroborated these results; in that study, the type of medical insurance was significantly associated with patients’ HRQoL, and low reimbursement coverage was identified as an independent predictor of poorer outcomes in HD patients [52]. Supplemental insurance improves HRQoL in HD patients by reducing financial burden and increasing access to care, with its effect varying according to the coverage and support provided by the country’s healthcare system.

Tobacco users scored an average of 3.72 units lower on the MCS compared to non-smokers, which was statistically significant. Studies from Korea and China identified smoking as a potential factor linked to lower MCS, which may be associated with increased anxiety, depression, and psychological distress [9,53]. In Iran, smoking was associated with impaired sleep quality, potentially mediating its adverse effects on mental health [54]. Similarly, in Saudi Arabia, smoking correlated with lower PCS and MCS scores among hospitalized CKD patients [55]. In contrast, a study from Turkey reported no difference in smoking prevalence between dialysis patients and nondialysis patients and reported no association with HRQoL [56]. Tobacco use is associated with reduced mental health in HD patients, but the magnitude of its effect may depend on cultural factors, social support, access to mental health services, and differences in healthcare systems across countries, which may explain the inconsistent findings in cross-country studies.

Age, sex, and comorbidities remained in the final multivariable regression model but did not exhibit any statistically significant associations with HRQoL scores. While several studies have identified older age as a significant predictor of reduced HRQoL [20,21,57], other evidence suggests that, among HD patients, this relationship may be influenced by psychological adaptation, disease severity, and chronicity, resulting in a nonlinear or nonsignificant association [50,58,59]. Findings on gender are similarly inconsistent, whereas some studies have reported lower HRQoL scores in females [21,58], others have reported no significant differences [50]. These discrepancies may partly reflect the potential moderating role of cultural factors, social roles, and family support. Furthermore, although some previous studies have suggested that comorbidities may be associated with lower HRQoL [21,27,60,61], our findings, which are consistent with some prior reports [62], revealed no significant associations. Several contextual factors may explain our null findings. First, the substantial physical and psychosocial burden of long-term HD may exert a dominant influence on HRQoL, thereby diminishing the relative contribution of demographic characteristics. In our sample, HRQoL scores were consistently low across age groups and between men and women, indicating that dialysis-related constraints may overshadow demographic differences. Second, more than half of the participants had two or more comorbidities, resulting in a relatively homogeneous clinical profile and limited variability across subgroups. This lack of variation reduces statistical power to detect adjusted associations. Additionally, the absence of detailed information on comorbidity severity may have introduced misclassification, attenuating potential true effects. Collectively, these factors likely contributed to the absence of significant associations in multivariable analyses.

In the present study, the highest frequency of “any problem” was observed in the P/D dimension (61.6%), followed by MO and A/D (both 54.7%), whereas the lowest frequency was reported in SC (25.6%). This pattern aligns with findings from the studies by Wasserfallen, Zhang, and Zyoud, which similarly reported the highest burden in P/D and A/D and the lowest in SC [20,21,63]. Furthermore, older patients (over 60 years), those with two or more comorbidities, illiterate individuals, and divorced or widowed participants reported significantly more problems across most or all dimensions. These findings suggest that targeted management of pain and anxiety/depression, along with focused support for older adults and patients with higher disease burden or lower socioeconomic status, may be crucial for improving HRQoL in this population.

### 4.2. Implications for practice and policy

The findings of this study present a multidimensional roadmap for enhancing the HRQoL of HD patients. Redesigning health education for individuals with lower literacy—incorporating visual and interactive approaches—may foster better understanding and more active participation in self-care. Strengthening psychological and social support for those who have lost a partner can not only reduce feelings of loneliness but also bolster emotional resilience. Flexible employment opportunities tailored to patients’ physical capacities, alongside economic support, may help sustain their social connections and everyday roles. Expanding supplemental insurance coverage and reducing direct treatment costs can ease financial strain while improving access to high-quality care. For patients with longer dialysis histories, regular monitoring and fatigue management programs can help preserve both physical and mental capacity. Finally, integrating smoking cessation counselling and services into routine care offers an opportunity to simultaneously improve physical and mental health outcomes.

### 4.3. Strengths and limitations

This study benefits from a relatively large and diverse sample of HD patients, the use of validated instruments for assessing HRQoL, and robust multivariable regression modelling. The inclusion of a broad range of demographic, socioeconomic, behavioral, and clinical variables also allowed for a more comprehensive evaluation of factors influencing HRQoL.

However, the cross-sectional design limits causal inference, and reliance on self-reported data may introduce recall or social desirability bias. Conducting the study within a single regional context may also reduce the generalizability of the findings. Despite these limitations, the results provide a basis for designing targeted interventions to improve the quality of life of HD patients. In addition, there is a potential for residual confounding due to unmeasured clinical and socio-economic factors, including comorbidity severity and socio-economic conditions, which may have attenuated the observed associations between age, sex, and the number of comorbidities and HRQoL.

## 5. Conclusion

This study highlights the multifaceted nature of HRQoLin HD patients, shaped by educational attainment, marital status, employment, supplemental insurance, dialysis duration, and smoking habits. These findings emphasize that, beyond clinical management, addressing social, economic, and behavioral factors is essential for improving patient outcomes. Tailored educational programs, enhanced psychosocial support, expanded insurance coverage, and lifestyle interventions could collectively contribute to better quality of life in this population. Further longitudinal research is warranted to confirm these associations and guide targeted strategies.

## Supporting information

S1 Table Mean scores of KDQoL SF subscales among study population. (DOCX)

S2 Table Statistical comparison of EQ-5D-5L dimension problems across demographic and clinical subgroups in study population. (DOCX)

S3 Table Multivariate linear regression analyses of factors associated with EQ-5D index, EQ-VAS, PCS, and MCS scores. (DOCX)
