# Supplementary material for: Health-related quality of life in patients on maintenance hemodialysis: Evidence from southern Iran using EQ-5D-5L and KDQOL-SF
Source: PLoS One. 2026 Feb 13;21(2):e0342155. doi: 10.1371/journal.pone.0342155 (PMC12904445; doi:10.1371/journal.pone.0342155)
Supplement: S1 Table — (DOCX) [file pone.0342155.s001.docx]

**S1 Table.** Mean Scores of KDQoL SF Subscales among

Study Population (n = 203).

| **Subscale** | **Mean ± SD** |
| --- | --- |
| Symptom problem list | 73.29 ± 16.35 |
| Effects of kidney disease | 58.85 ± 19.83 |
| Burden of kidney disease | 39.66 ± 25.83 |
| Work status | 30.79 ± 32.63 |
| Cognitive function | 67.85 ± 18.32 |
| Quality of social interaction | 68.80 ± 21.00 |
| Sexual function | 77.63 ± 23.57 |
| Sleep | 59.45 ± 22.43 |
| Social support | 79.72 ± 23.41 |
| Dialysis staff encouragement | 82.57 ± 21.87 |
| Patient satisfaction | 56.08 ± 29.32 |
| Overall Health | 62.51 ± 23.38 |
| Physical functioning | 55.54 ± 31.13 |
| Role physical | 68.35 ± 36.12 |
| Pain | 62.00 ± 27.66 |
| General health | 45.44 ± 20.76 |
| Energy fatigue | 49.11 ± 23.55 |
| Social function | 66.93 ± 26.74 |
| Role emotional | 54.68 ± 40.27 |
| Emotional well being | 56.51 ± 20.32 |

KDQoL-SF: Kidney Disease Quality of Life Short Form;

SD: Standard deviation.
