# Supplementary material for: Health-related quality of life in patients on maintenance hemodialysis: Evidence from southern Iran using EQ-5D-5L and KDQOL-SF
Source: PLoS One. 2026 Feb 13;21(2):e0342155. doi: 10.1371/journal.pone.0342155 (PMC12904445; doi:10.1371/journal.pone.0342155)
Supplement: S2 Table — (DOCX) [file pone.0342155.s002.docx]

**S2 Table.** Statistical Comparison of EQ-5D-5L Dimension Problems Across Demographic and Clinical Subgroups in Study Population (n = 203).

| **Variables** | **Number and percentage with “any problem”** | | | | |
| --- | --- | --- | --- | --- | --- |
|  | **MO**  n(%) | **SC**  n(%) | **UA**  n(%) | **P/D**  n(%) | **A/D**  n(%) |
| Total | 111 (54.68) | 52 (25.62) | 99 (48.77) | 125 (61.58) | 111 (54.68) |
| Age |  |  |  |  |  |
| <40 | 9 (23.68) | 5 (13.16) | 12 (31.58) | 15 (39.47) | 22 (57.89) |
| 40-60 | 37 (45.68) | 9 (11.11) | 33 (40.74) | 46 (56.79) | 41 (50.62) |
| >60 | **65 (77.38)** | **38 (45.24)** | **54 (64.29)** | 64 (76.19) | 48 (57.14) |
| Gender |  |  |  |  |  |
| Male | 51 (47.66) | 24 (22.43) | 49 (45.79) | 61 (57.01) | 55 (51.4) |
| Female | **60 (62.5)** | 28 (29.17) | 50 (52.08) | 64 (66.67) | 56 (58.33) |
| Marital Status |  |  |  |  |  |
| Single | 9 (50) | 4 (22.22) | 7 (38.89) | 9 (50) | 7 (38.89) |
| Married | 85 (50.9) | 35 (20.96) | 77 (46.11) | 100 (59.88) | 89 (53.29) |
| Divorced/widowed | **17 (94.44)** | **13 (72.22)** | **15 (83.33)** | **16 (88.89)** | **15 (83.33)** |
| Education |  |  |  |  |  |
| Illustrate | **48 (87.27)** | **32 (58.18)** | **40 (72.73)** | 41 (74.55) | 35 (63.64) |
| <6 classes | 29 (52.73) | 9 (16.36) | 28 (50.91) | 34 (61.82) | 28 (50.91) |
| 6-12 classes | 29 (38.67) | 10 (13.33) | 27 (36) | 42 (56) | 42 (56) |
| >12 classes | 5 (27.78) | 1 (5.56) | 4 (22.22) | 8 (44.44) | 6 (33.33) |
| Occupation |  |  |  |  |  |
| Housekeeper | 53 (61.63) | 24 (27.91) | 46 (53.49) | 56 (65.12) | 50 (58.14) |
| Employed | 14 (31.11) | 3 (6.67) | 16 (35.56) | 26 (57.78) | 25 (55.56) |
| Disabled/Retried | **37 (66.07)** | **23 (41.07)** | 30 (53.57) | 35 (62.5) | 28 (50) |
| Unemployment | 7 (43.75) | 2 (12.5) | 7 (43.75) | 8 (50) | 8 (50) |
| Residence |  |  |  |  |  |
| Rural | 32 (54.24) | 18 (30.51) | 31 (52.54) | 39 (66.1) | **40 (67.8)** |
| City | 79 (54.86) | 34 (23.61) | 68 (47.22) | 86 (59.72) | 71 (49.31) |
| Sup insurance |  |  |  |  |  |
| No | 55 (52.38) | 29 (27.62) | 50 (47.62) | 64 (60.95) | 63 (60) |
| Yes | 56 (57.14) | 23 (23.47) | 49 (50) | 61 (62.24) | 48 (48.98) |
| Tobacco use |  |  |  |  |  |
| No | 65 (52.42) | 28 (22.58) | 59 (47.58) | 72 (58.06) | 59 (47.58) |
| Former user | 32 (60.38) | 18 (33.96) | 29 (54.72) | 37 (69.81) | **37 (69.81)** |
| Yes | 14 (53.85) | 6 (23.08) | 11 (42.31) | 16 (61.54) | 15 (57.69) |
| Comorbidity |  |  |  |  |  |
| 0 | 10 (35.71) | 4 (14.29) | 7 (25) | 15 (53.57) | 14 (50) |
| 1 | 29 (40.85) | 10 (14.08) | 30 (42.25) | 37 (52.11) | 39 (54.93) |
| >=2 | **72 (69.23)** | **38 (36.54)** | **62 (59.62)** | **73 (70.19)** | 58 (55.77) |
| Main cause of CKD |  |  |  |  |  |
| Not knowing/Unknown | 18 (40.91) | 8 (18.18) | 14 (31.82) | 30 (68.18) | 24 (54.55) |
| Diabetes | 34 (56.67) | 16 (26.67) | 32 (53.33) | 36 (60) | 31 (51.67) |
| Hypertension | 43 (61.43) | 20 (28.57) | **41 (58.57)** | 44 (62.86) | 43 (61.43) |
| Others | 16 (55.17) | 8 (27.59) | 12 (41.38) | 15 (51.72) | 13 (44.83) |
| Kidney transplant |  |  |  |  |  |
| No | 107 (55.73) | 49 (25.52) | 94 (48.96) | 120 (62.5) | 105 (54.69) |
| Yes | 4 (36.36) | 3 (27.27) | 5 (45.45) | 5 (45.45) | 6 (54.55) |
| Hospitalization |  |  |  |  |  |
| No | 25 (45.45) | 6 (10.91) | 21 (38.18) | 31 (56.36) | 26 (47.27) |
| Yes | 86 (58.11) | **46 (31.08)** | 78 (52.7) | 94 (63.51) | 85 (57.43) |
| Dialysis duration (yrs) |  |  |  |  |  |
| <5 | 79 (51.97) | 36 (23.68) | 70 (46.05) | 91 (59.87) | 82 (53.95) |
| ≥5 | 32 (62.75) | 16 (31.37) | 29 (56.86) | 34 (66.67) | 29 (56.86) |
| Blood pressure |  |  |  |  |  |
| Normal | 41 (48.24) | 17 (20) | 39 (45.88) | 47 (55.29) | 41 (48.24) |
| Semi-Normal | 22 (62.86) | 12 (34.29) | 16 (45.71) | 24 (68.57) | 19 (54.29) |
| High | 48 (57.83) | 23 (27.71) | 44 (53.01) | 54 (65.06) | 51 (61.45) |

MO: Mobility; SC: Self-care; UA: Usual activities; P/D: Pain/Discomfort; A/D: Anxiety/Depression; Sup insurance: Supplemental Insurance. "Any problem" is defined as reporting level 2 to 5 in each EQ-5D-5L dimension. P-values are derived from Chi-square test or Fisher’s exact test, depending on expected cell counts. **Bold values** p < 0.05.
