# Supplementary material for: Health-related quality of life in patients on maintenance hemodialysis: Evidence from southern Iran using EQ-5D-5L and KDQOL-SF
Source: PLoS One. 2026 Feb 13;21(2):e0342155. doi: 10.1371/journal.pone.0342155 (PMC12904445; doi:10.1371/journal.pone.0342155)
Supplement: S3 Table — (DOCX) [file pone.0342155.s003.docx]

**S3 Table.** Multivariate Linear Regression Analyses of Factors Associated with EQ-5D Index, EQ-VAS, PCS, and MCS Scores (n=203).

| **Variables** | **EQ-5D-Index**  **β (95% CI)** | **EQ-VAS**  **β (95% CI)** | **PCS**  **β (95% CI)** | **MCS**  **β (95% CI)** |
| --- | --- | --- | --- | --- |
| Age | -0.01 (-0.01 to 0.00) | -0.11 (-0.55 to 0.32) | -0.07 (-0.21 to 0.07) | 0.06 (-0.06 to 0.18) |
| Gender (Male) | **Reference** | | | |
| Female | -0.15 (-0.34 to 0.03) | -17.89 (-40.98 to 5.21) | -3.51 (-7.15 to 0.13) | -4.45 (-9.49 to 0.59) |
| Residence (Rural) | **Reference** | | | |
| City | -0.02 (-0.17 to 0.13) | -1.28 (-9.63 to 7.07) | 0.44(-2.44 to 3.31) | 0.27 (-3.39 to 3.93) |
| Marital Status (Single) | **Reference** | | | |
| Married | 0.11 (-0.26 to 0.47) | 0.29 (-14.82 to 15.41) | -0.17 (-4.49 to 4.14) | -0.04 (-8.44 to 8.36) |
| Divorced/widowed | -0.31 (-0.74 to 0.12) | -13.75 (-34.36 to 6.96) | -4.54(-10.71 to 1.63) | -4.88 (-14.62 to 4.85) |
| Occupation (housekeeper) | **Reference** | | | |
| Employed | -0.11 (-0.29 to 0.08) | -23.57 (-50.02 to 2.88) | -0.65 (-5.39 to 4.09) | -4.82 (-11.23 to 1.58) |
| Disabled/Retried | -0.18 (-0.39 to 0.04) | **-26.14 (-50.11 to -2.17)** | -3.22(-7.36 to 0.93) | -2.93 (-7.54 to 1.68) |
| Unemployment | -0.23 (-0.39 to 0.04) | -21.24 (-44.80 to 2.33) | **-4.72 (-8.65 to -0.79)** | -6.17 (-13.29 to 0.96) |
| Education (Illustrate) | **Reference** | | | |
| <6 class | **0.22 (0.01 to 0.43)** | 5.68 (-6.93 to 18.28) | 2.14 (-2.24 to 6.52) | 0.02 (-3.95 to 4.00) |
| 6-12 class | 0.20 (-0.04 to 0.44) | 7.98 (-7.35 to 23.31) | 2.32(-2.66 to 7.30) | -0.91(-5.71 to 3.89) |
| >12 class | 0.27 (-0.01 to 0.56) | 10.43 (-10.66 to 31.52) | -1.55 (-8.04 to 4.94) | -0.02 (-5.81 to 5.76) |
| Sup insurance (No) | **Reference** | | | |
| Yes | 0.03 (-0.10 to 0.17) | 5.10 (-4.49 to 14.69) | **2.62(0.34 to 4.89)** | 1.88 (-1.30 to 5.05) |
| Tobacco use (No) | **Reference** | | | |
| Former user | -0.02 (-0.17 to 0.14) | 5.21(-4.59 to 15.02) | -0.81(-3.99 to 2.37) | -1.56 (-4.79 to 1.67) |
| Yes | -0.03 (-0.20 to 0.13) | 0.92(-10.96 to 12.79) | -2.86(-7.25 to 1.53) | -3.32 (-7.43 to 0.78) |
| Comorbidity(0) | **Reference** | | | |
| Comorbidity (1) | 0.06 (-0.13 to 0.25) | -1.45(-12.30 to 9.39) | -1.18 (-5.71 to 3.34) | 3.48 (-1.15 to 8.10) |
| Comorbidity (>=2 | -0.07 (-0.28 to 0.14) | -6.63(-17.60 to 4.34) | -3.61 (-8.48 to 1.26) | 2.99 (-1.68 to 7.66) |
| Dialysis Duration | **-0.17 (-0.34 to -0.01)** | -0.71 (-12.86 to 11.44) | 1.63 (-1.25 to 4.51) | -2.47 (-6.08 to 1.15) |
| Hospitalization (No) | **Reference** | | | |
| Yes | -0.03 (-0.16 to 0.10) | -5.37 (-14.57 to 3.83) | -1.37 (-4.47 to 1.72) | -0.24 (-3.94 to 3.46) |
| Kidney transplant (No) | **Reference** | | | |
| Yes | -0.02 (-0.35 to 0.31) | -10.03(-29.70 to 9.64) | -3.40 (-8.68 to 1.88) | 3.86 (-4.83 to 12.55) |
| Kt/V index | -0.01 (-0.31 to 0.28) | -1.30(-14.04 to 11.43) | -0.60(-3.18 to 1.99) | 2.05 (-2.87 to 6.96) |
| Hemoglobin | 0.01 (0.00 to 0.30) | 0.59(-0.51 to 1.70) | 0.10(-0.33 to 0.54) | -0.05 (-0.63 to 0.52) |
| Phosphorus | 0.001 (-0.04 to 0.04) | -0.06(-2.19 to 2.07) | 0.54 (-0.27 to 1.35) | -0.35 (-1.41 to 0.70) |

EQ-5D Index: EQ-5D-5L Utility Index Score; EQ-VAS: EuroQol Visual Analogue Scale; PCS: SF-12 Physical Component Summary Score; MCS: SF-12 Mental Component Summary Score; Sup insurance: Supplemental Insurance; CI: Confidence interval; **Bold values** p < 0.05.
